# Leprosy

**Published:** 1902-06-28

**Authors:** 


### LEPROSY.

At the adjourned discussion on leprosy at the Royal Medical and Chirurgical Society nearly all the speakers favoured the hypothesis of contagion as the means by which the disease is spread, and opposed the suggestion that it is caused by the use of fish as a food. Moreover, there was a general leaning towards the enforcement of segregation of lepers as being the best means of stopping the spread of the disease. It must be confessed, however, that, as Mr. Hutchinson said in his reply, all the speakers passed over the difficulties of the contagion theory. The most interesting of the communications was that made by Dr. Hansen, of Bergen. He said that the bacillus could be cultivated, but only with considerable difficulty. His experience in Norway supported the belief that all cases could be explained on the theory of contagion. He went on to say that since 1856, at which time there were 2,870 lepers in Norway, the lepers had been isolated, and that since then there had been a steady decline in the number of cases until at the present time there were only about 400 cases. He added, however—and this takes away greatly from the force of what he had just said about the advantages of isolation—that in America none of the Norwegian immigrants, of whom there were an immense number, had developed leprosy. This, he thought, proved that the disease was not hereditary, but it would seem equally to prove that leprosy is to be stopped, not by segregation, but by improving the hygienic surroundings of the people.

